# Micro-computed tomography (micro-CT) for the assessment of myocardial disarray, fibrosis and ventricular mass in a feline model of hypertrophic cardiomyopathy

**DOI:** 10.1038/s41598-020-76809-5

**Published:** 2020-11-19

**Authors:** Jose Novo Matos, Patricia Garcia-Canadilla, Ian C. Simcock, J. Ciaran Hutchinson, Melanie Dobromylskyj, Anna Guy, Owen J. Arthurs, Andrew C. Cook, Virginia Luis Fuentes

**Affiliations:** 1 Clinical Sciences and Services, Royal Veterinary College, London, UK; 2 Institut D’Investigacions Biomèdiques August Pi I Sunyer, Barcelona, Spain; 3 Institute of Cardiovascular Science, University College London, London, UK; 4 Department of Histopathology, Great Ormond Street Hospital for Children, NHS Foundation Trust, London, UK; 5 Department of Radiology, Great Ormond Street Hospital for Children, NHS Foundation Trust, London, UK; 6 National Institute for Health Research Great Ormond Street Hospital Biomedical Research Centre, London, UK; 7 Finn Pathologists, Norfolk, UK

**Keywords:** Cardiac hypertrophy, Cardiovascular biology

## Abstract

Micro-computed tomography (micro-CT) is a high-resolution imaging modality that provides accurate tissue characterization. Hypertrophic cardiomyopathy (HCM) occurs as a spontaneous disease in cats, and is characterized by myocardial hypertrophy, disarray and fibrosis, as in humans. While hypertrophy/mass (LVM) can be objectively measured, fibrosis and myocyte disarray are difficult to assess. We evaluated the accuracy of micro-CT for detection and quantification of myocardial disarray and fibrosis by direct comparison with histopathology. 29 cat hearts (12 normal and 17 HCM hearts) underwent micro-CT and pathologic examination. Myocyte orientation was assessed using structure tensor analysis by determination of helical angle (HA), fractional anisotropy (FA) and myocardial disarray index (MDI). Fibrosis was segmented and quantified based on comparison of gray-scale values in normal and fibrotic myocardium. LVM was obtained by determining myocardial volume. Myocardial segments with low FA, low MDI and disruption of normal HA transmural profile on micro-CT were associated with myocardial disarray on histopathology. FA was consistently lower in HCM than normal hearts. Assessment of fibrosis on micro-CT closely matched the histopathologic evaluation. LVM determined by micro-CT was higher in HCM than normal hearts. Micro-CT can be used to detect and quantify myocardial disarray and fibrosis and determine myocardial mass in HCM.

## Introduction

Hypertrophic cardiomyopathy (HCM) is a myocardial disease characterized by a hypertrophied left ventricle (LV) in the absence of abnormal loading conditions^1,2^. HCM is a common spontaneous cardiac disease of cats^3^, and the clinical and histopathologic features closely mimic those of the human disease^2,4^.

Micro-computed tomography (micro-CT) is an imaging modality that enables high resolution imaging with three-dimensional (3D) micrometer resolution, and is used in the postmortem setting or for extracted organs^5–9^. Micro-CT has been shown to be highly accurate in cardiac phenotyping in both humans and mice^8–10^, with superior assessment of myocardial morphology and coronary artery anatomy compared with dissecting microscopy^9^. Iodine-enhanced micro-CT differentiates cardiac tissue types based on differential attenuation of X-rays^6,7,11^, and allows assessment of the orientation of aggregated myocytes in canine and human hearts^6,7,12^.

The capability of micro-CT to provide 3D virtual slicing of the whole heart allows an extensive cardiac assessment that has potential application as part of routine cardiac postmortem examinations. Micro-CT would be especially useful in conditions where lesions are irregularly distributed, as an imaging guide to tissue sampling, thereby improving on the diagnostic yield of conventional histopathology.

HCM has traditionally been diagnosed with histopathology based on the presence of 3 main features: left ventricular hypertrophy (LVH); myocardial disarray; and fibrosis^13–15^. Diagnosis of HCM can be challenging, as these cardinal features may be focal and do not necessarily match areas of LVH^16^, and therefore can be missed without extensive sampling^15^. Moreover, HCM is characterized by a spectrum of morphologic changes and a highly heterogeneous phenotype, with variable LVH distribution. Isolated LVH can be caused by HCM phenocopies, so accurate quantification of disarray and fibrosis is important for postmortem diagnostic accuracy.

Our aim was to evaluate the ability of micro-CT to detect the principal features of HCM by comparison with histopathology, and to explore the use of micro-CT to quantify myocardial fibrosis, myocyte disarray and LV mass (LVM).

## Results

Twenty-nine hearts were included in the study (12 normal, 17 HCM). Mean scan duration was 75 min for the whole heart and 150 min for each LV section.

Micro-CT mean cohort voxel size was 24 µm for the whole heart and 16 µm for the LV section scans. LVM determination was possible in 28/28 available whole heart scans. Quantification of LV fibrosis was performed in 13/17 HCM hearts, as poor myocardial detail in 4/17 scans hindered accurate fibrosis segmentation. Analysis of the orientation of aggregated myocytes was possible in 16/29 hearts, with inadequate image quality precluding analysis in the remaining hearts. The cause for reduced image quality on micro-CT in these hearts was unclear.

### Determination of LVM

Actual LVM from 13 hearts (6 normal, 7 HCM) was greater (7.4 ± 2.5 g) than LVM obtained from M1-Fiji (5.9 ± 2.1 g) or M2-Imaris (6.0 ± 2.2 g), p < 0.0001. Both micro-CT methods yielded LVM values that were consistently lower than aLVM, with an average bias of 1.5 g and 95% limits of agreement between 0.08–3.0 (M1-Fiji) and 0.05–2.8 (M2-Imaris). LVM (M2-Imaris) was greater (7.8 ± 2.6 g) in 16 HCM hearts than in 12 normal hearts (4.9 ± 1.1 g), p = 0.001.

### Quantification of myocardial fibrosis

**Figure 1:**
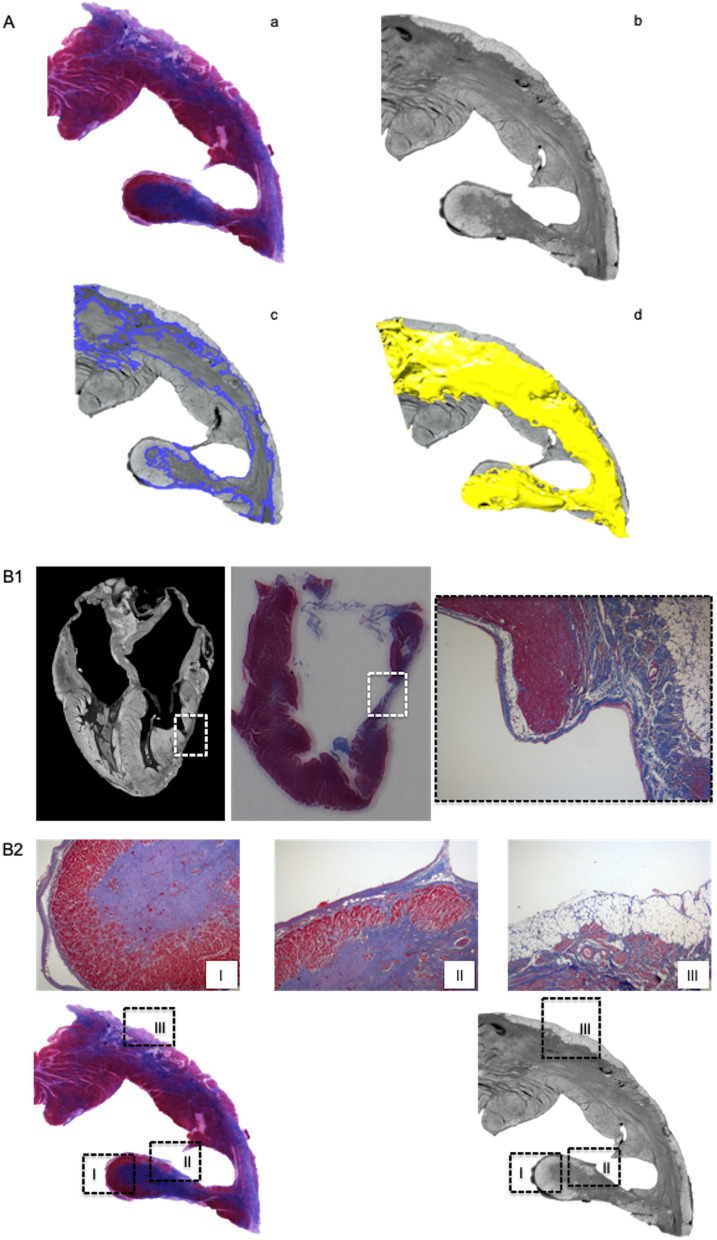
Matched ventricular sections on micro-CT and histopathology in feline hearts with HCM. (**A**) Segmentation was performed based on gray-scale gradient. a. Section stained with Masson’s trichrome (blue represents fibrosis); b. matched micro-CT image; c. segmentation based on differential gray-scale between normal myocardium versus fibrosis; d. 3D surface (yellow) representing fibrotic tissue in the whole LV section. (**B**) Matched longitudinal (**B1**) and transverse (**B2**) ventricular sections in 2 feline hearts with HCM on micro-CT and histopathology. Hypodense lesions on micro-CT corresponded to fibrosis on histology. Histology slides stained with Masson’s trichrome (**B2** I–III; × 10 magnification).

Micro-CT images of the LV section datasets were matched to the corresponding histology slide stained with Masson’s trichrome. Replacement myocardial fibrosis on histology appeared as hypodense areas on micro-CT images (Fig. 1), allowing segmentation and quantification of fibrosis based on gray-scale gradient. Conversely, interstitial fibrosis was not detected on micro-CT.

**Figure 2:**
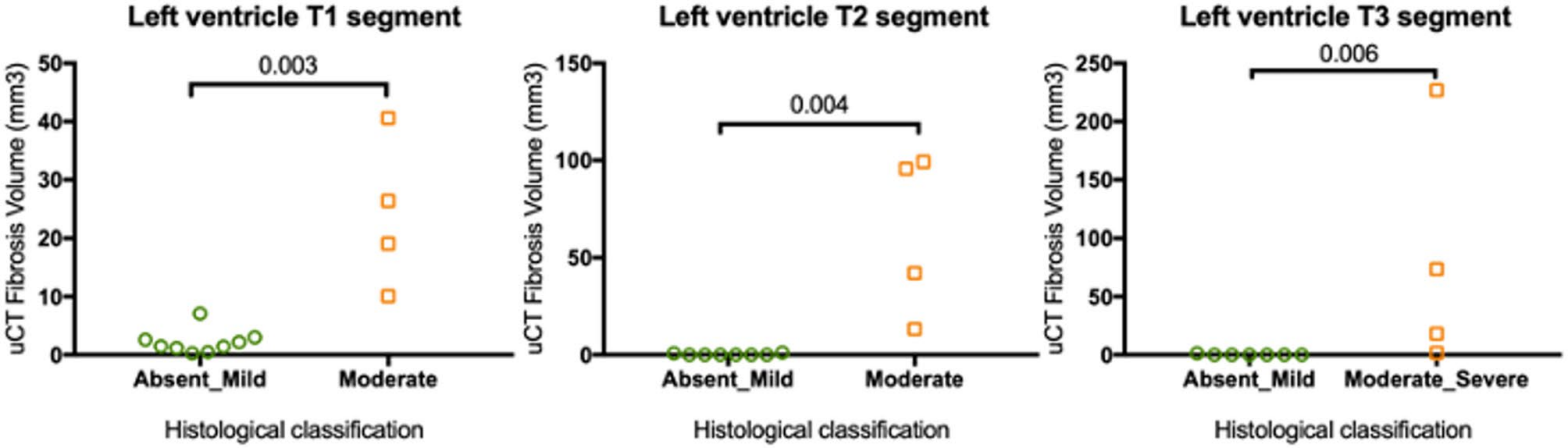
Relationship between semi-quantitative histologic score and micro-CT quantification of replacement myocardial fibrosis in LV sections T1 (**A**), T2 (**B**) and T3 (**C**).

Replacement fibrosis in the LV section T1 was classified as absent or mild in 9 hearts, and moderate in 4 hearts based on histopathology. The corresponding median fibrosis volume determined by micro-CT was 1.47 mm^3^ [0.27–7.1] and 22.8 mm^3^ [10.1–40.6] respectively (p = 0.003). In LV section T2, 8 hearts had absent or mild fibrosis and 4 had moderate fibrosis, which corresponded to fibrosis volumes of 0.11 mm^3^ [0–1.2] and 69 mm^3^ [13.3–99.3], respectively (p = 0.004). In LV section T3, 7 hearts had absent or mild fibrosis and 4 moderate or severe fibrosis, with fibrosis volumes of 0.01 mm^3^ [0–1.4] and 46 mm^3^ [1.7–227], respectively (p = 0.006) (Fig. 2). The median fibrosis volume was higher in the basilar LV section (T1) in comparison with the apical section (T3) (p = 0.03) (T1 3.1 [0.3–41] mm^3^, T2 0.9 [0–99] mm^3^, T3 0.1 [0–227] mm^3^). Mean volume of fibrosis determined in the whole LV of 13 HCM hearts was 9.5 mm^3^ [1.3–1184].

### Quantification of the orientation of aggregated myocytes

**Figure 3:**
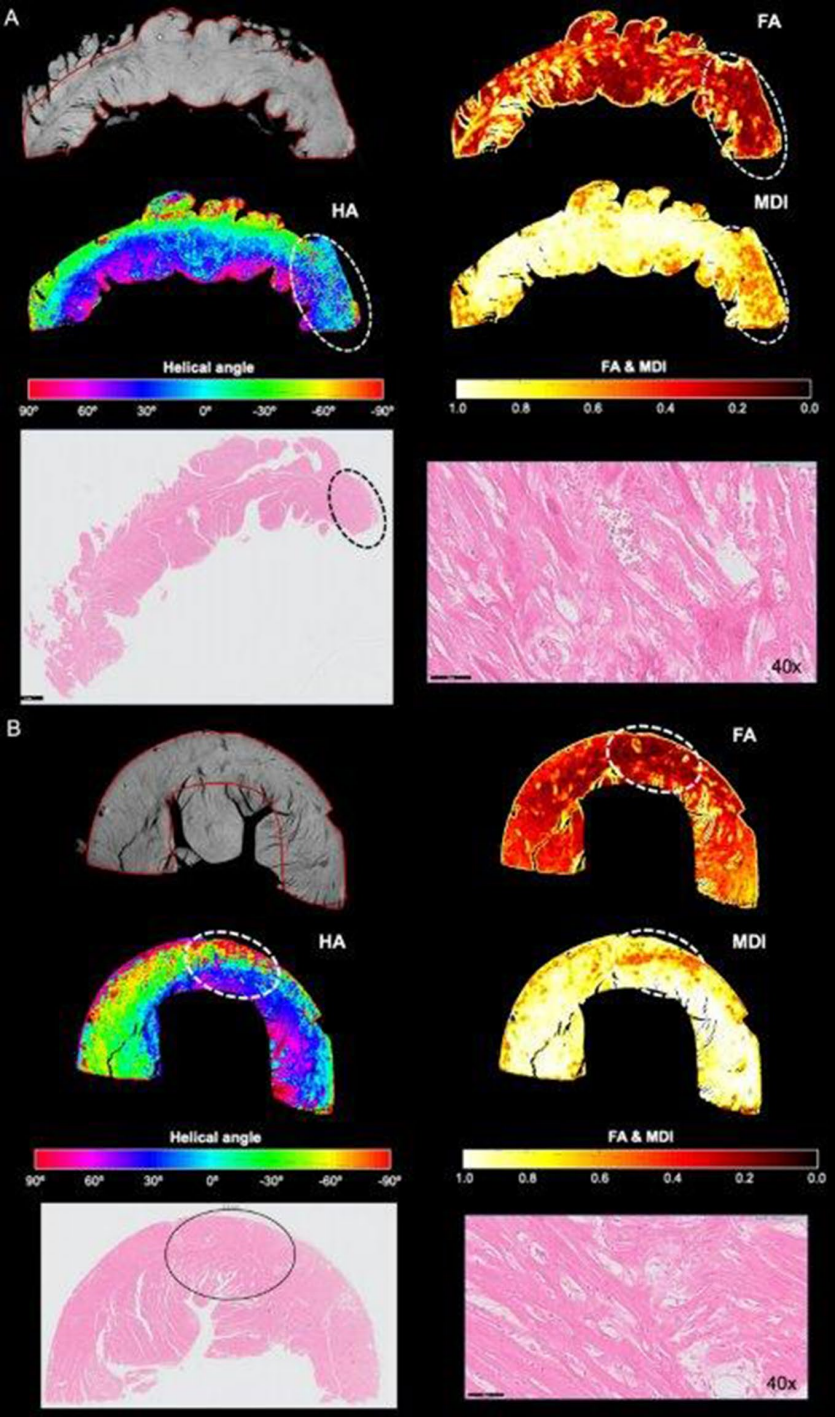
Matched transverse ventricular section (T2 section) in 2 feline hearts with HCM (**A**,**B**) on micro-CT and histopathology. Helical angle (HA), fractional anisotropy (FA) and myocardial disarray index (MDI) colored maps showing areas of disarray on micro-CT corresponding to disarray on histology. In HA colored map, disarray is seen as areas where there is a disruption of the smooth transmural profile of HA: negative angles in the epicardium (light green colored) to positive in the endocardium (pink colored). In FA and MDI maps, high FA or MDI (values close to 1, yellow/white colored) suggest negligible disarray, while low FA or MDI (near 0, red/black colored) represents disarray. Circled area, area of marked disarray; H&E, hematoxylin and eosin staining.

The HA, FA, MDI and percentage of circumferentially arranged myocytes were determined in the LV T2 sections of 16 hearts (5 normal, 11 HCM). T2 section colored maps of the different orientation variables were matched to the corresponding histology slide. Myocardial segments with low FA, low MDI and loss/disruption of the normal HA transmural profile on HA plots corresponded to areas of myocardial disarray on histology (Fig. 3).

**Figure 4:**
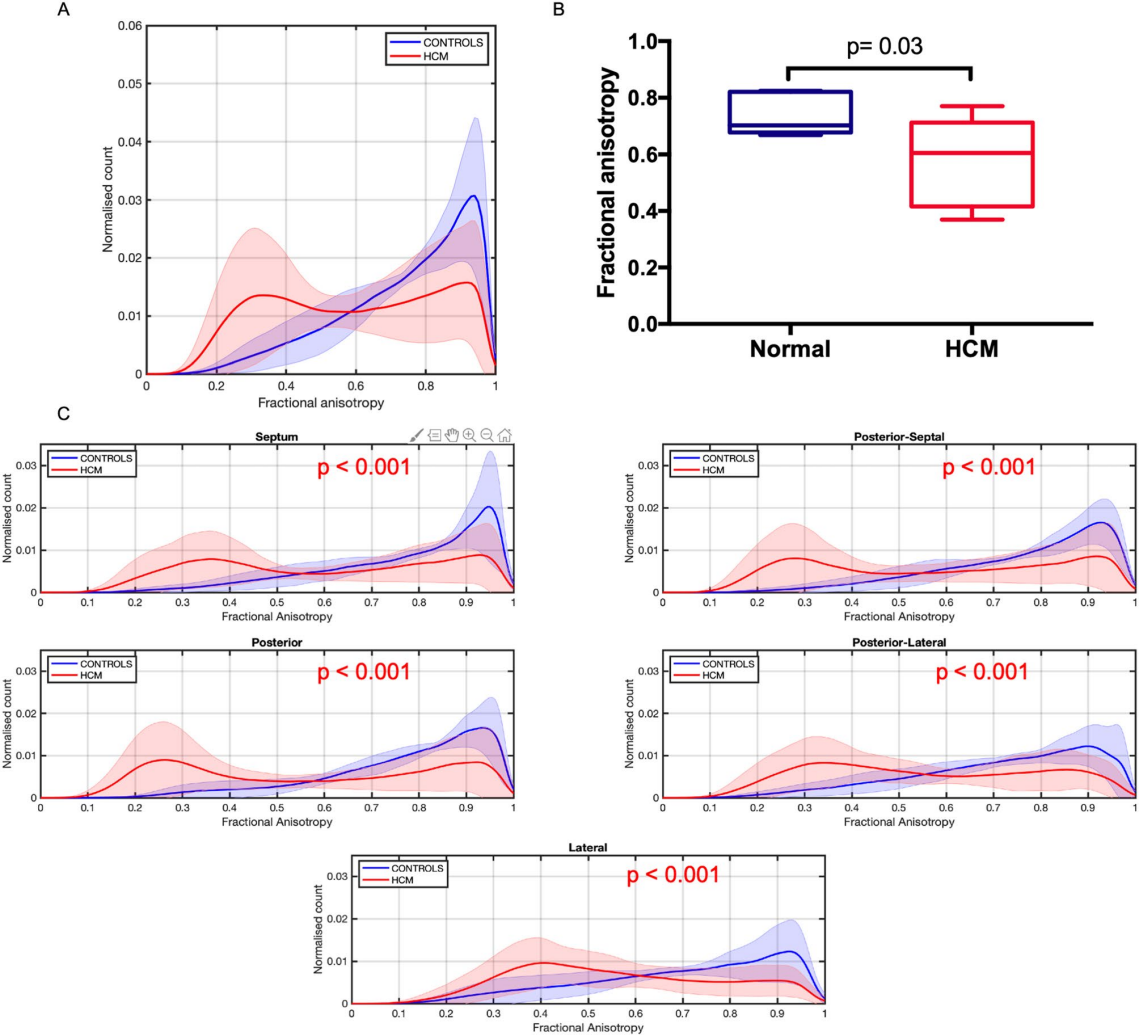
Fractional anisotropy (FA). (**A**) Histogram showing overall FA distribution in normal (controls, blue) vs HCM hearts (HCM, red). (**B**) Mean FA in the whole LV T2 section in normal and HCM hearts. (**C**) FA distribution in normal and HCM hearts in 5 different LV segments. Lines: group means; Ribbon: ± SD.

Overall FA distribution in the LV T2 section and across each of the 5 LV segments was different between normal and HCM hearts (Fig. 4). FA distribution in normal hearts peaked at values close to 1, while HCM hearts had lower FA values with a bimodal distribution characterized by a larger peak around 0.2–0.4. Mean FA of the whole LV T2 section was also lower in HCM hearts (0.57 ± 0.15 versus 0.74 ± 0.08, p = 0.03) (Fig. 4), but there was no difference in mean FA within LV segments.

**Figure 5:**
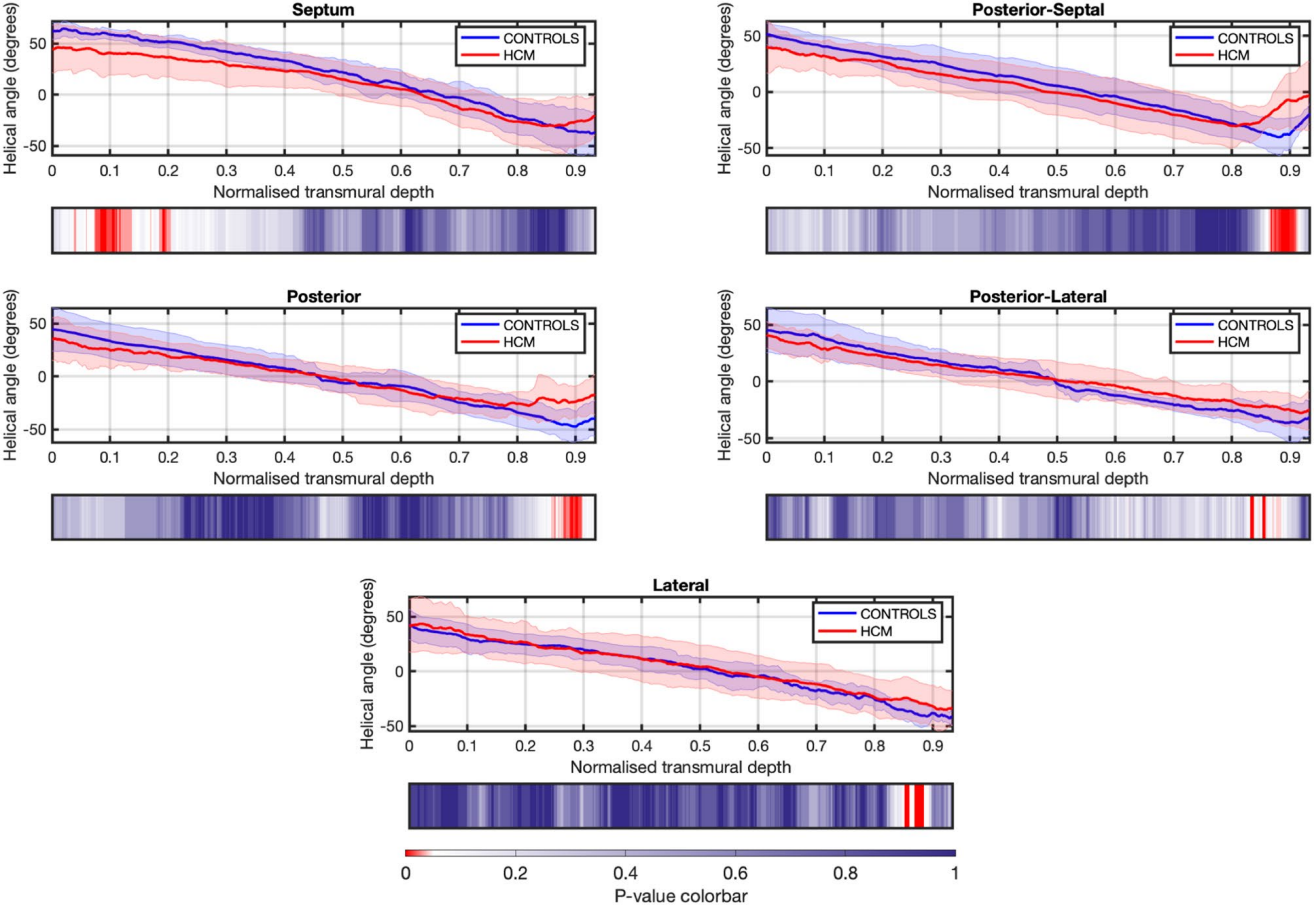
Transmural helical angle (HA) profile in 5 LV segments in normal (controls) and HCM hearts. Angles change gradually across the LV wall depth from around + 50° in the endocardium to − 50° in the epicardium or right-sided endocardium, reflecting the typical counterclockwise rotation of HA from positive angulation in the endocardium to negative angulation in the epicardium. Lines: group means; Ribbon: ± SD.

The overall HA distribution was similar between groups across the T2 section and 5 LV segments, although the mean HA transmural profiles differed between normal and HCM hearts in the endocardial/sub-endocardial area of the septum and the epicardial/sub-epicardial area in the posterior-septal, posterior, posterior-lateral and lateral LV segments (Fig. 5). This was also shown by less steep HA transmural profiles as demonstrated by the lower slopes (B1) of the linear regression fits of the HA transmural profiles between normal and HCM hearts (− 20.1 (3.8) versus − 13.2 (4.1), p < 0.001).

**Figure 6:**
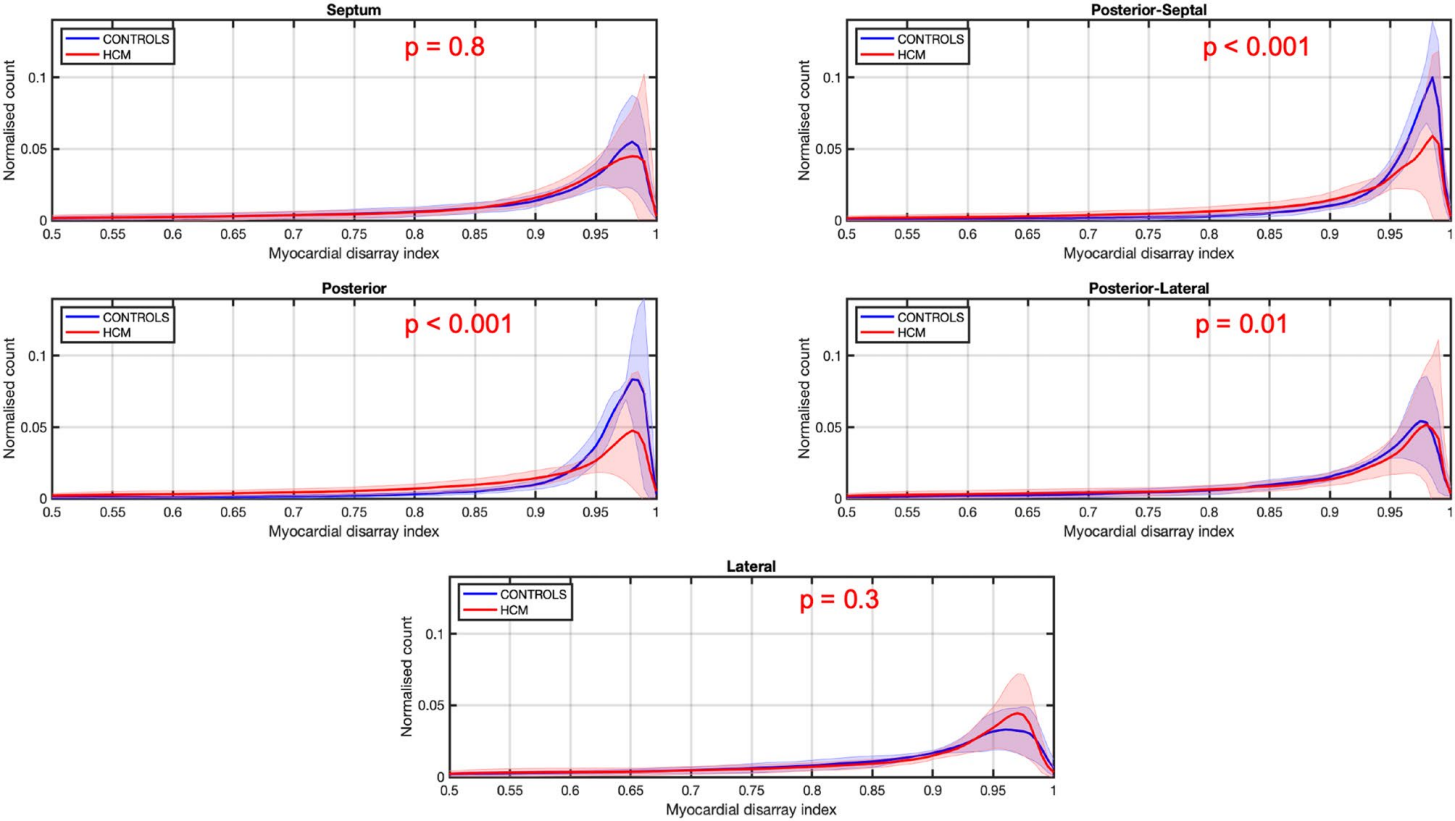
Averaged myocardial disarray index (MDI) distribution in normal and HCM hearts in 5 LV segments. Lines: group means; Ribbon: ± SD.

The overall MDI distribution was similar between groups when the whole LV T2 section was assessed, although MDI was markedly lower in HCM hearts in the posterior-septal and posterior segments (Fig. 6).

There was no difference in the percentage of circumferential arranged myocytes between normal and HCM hearts, and no correlation between FA or MDI and heart weight, aLVM and fibrosis.

## Discussion

We used contrast-enhanced micro-CT to scan feline hearts with and without HCM to quantify the key features for the diagnosis of HCM: LVM, the extent of fibrosis and abnormalities of the orientation of aggregated myocytes^14,15^.

### Left ventricular mass

Left ventricular hypertrophy or increased LV mass is the principal feature of HCM^1^. Whole heart weight is the standard pathologic surrogate measure of LVH, despite including the atria, great arteries and right ventricle. Postmortem imaging can provide a more accurate measure of LVH^17^, and our study showed that LVM can be determined by micro-CT. Measurements using the 2 micro-CT methods were comparable and highly consistent. Although micro-CT underestimated aLVM by approximately 20%, this may be attributable to mass determination calculation factors.

The direct measurements of LV weight (aLVM) included residual blood clots in the ventricular chamber, epicardial fat, and the mitral and aortic valves, which were excluded from the ROIs defined to calculate LVM on micro-CT and could potentially account for the observed differences. Although micro-CT methods yielded consistently lower LVM values, micro-CT still correctly differentiated normal from HCM hearts based on LVM, and is a potentially useful imaging tool for postmortem LVM estimation.

### Myocardial fibrosis

HCM is typically characterized by interstitial and replacement fibrosis^15^. In our micro-CT scans, replacement fibrosis was readily identified as hypodense areas. Iodine is believed to enhance myocardial contrast in postmortem specimens by becoming immobilized within glycogen in myocytes, which causes a greater X-ray attenuation of myocardium than fibrous tissue^18^. Fibrous tissue is therefore seen as darker areas on micro-CT (hypodense). Conversely, with in vivo clinical MRI and CT imaging, iodinated contrast agents accumulate in areas of fibrosis, as there is increased extracellular space due to myocyte loss. By retaining contrast, fibrotic areas are therefore seen with higher density/intensity in clinical CT/MRI^19,20^, which is the opposite of what is seen with micro-CT. As with clinical CT, we were not able to detect interstitial fibrosis with micro-CT^20^. Even with MRI, late gadolinium enhancement mainly detects replacement fibrosis^19^. The quantitative assessment of replacement fibrosis on micro-CT closely matched the semi-quantitative histopathologic evaluation.

### Myocyte orientation

**Figure 7:**
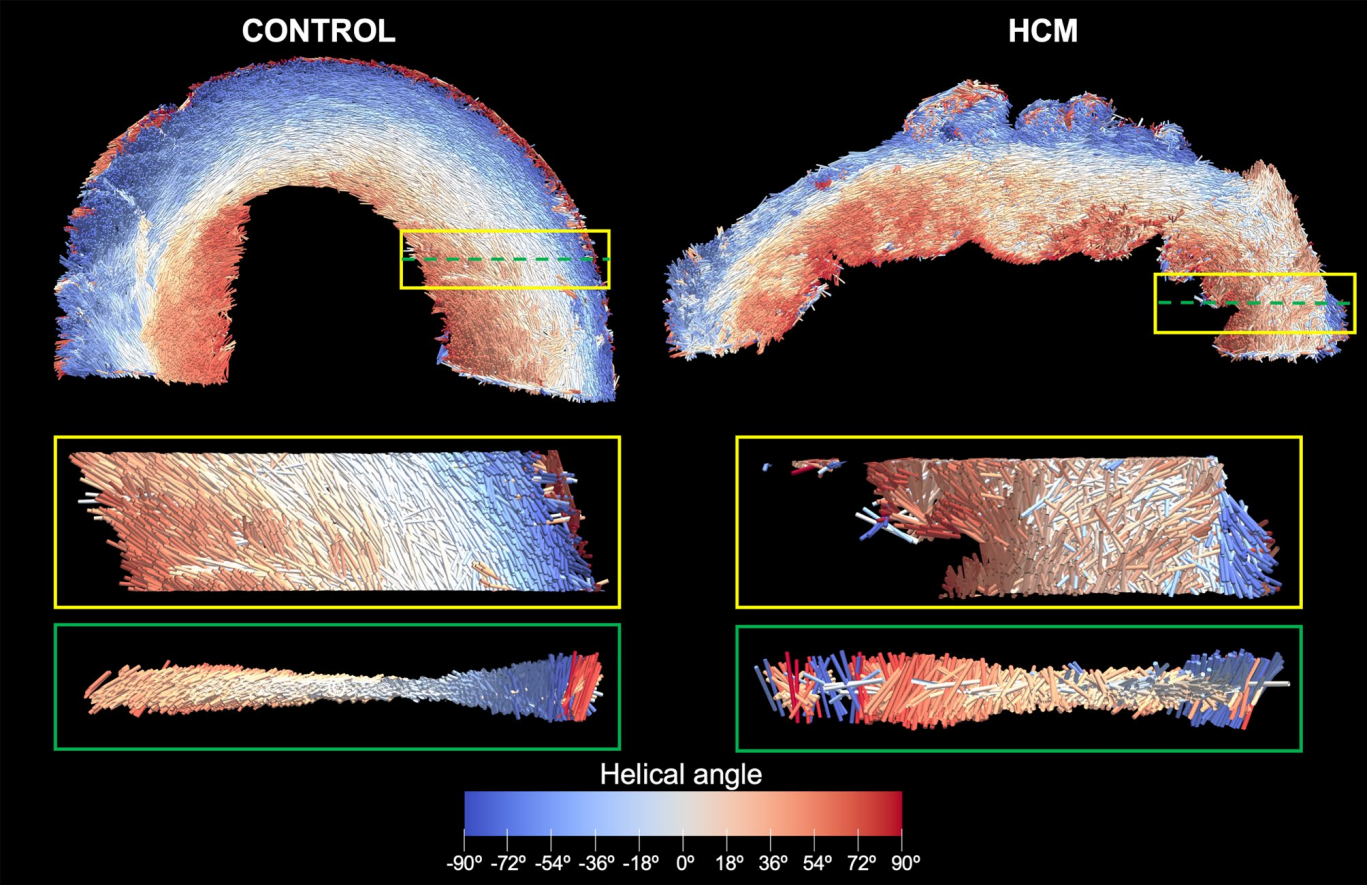
3D representation of the tertiary eigenvector representing the longitudinal direction of aggregated myocytes for a single mid-LV slice in a control and HCM heart. The insets represent magnified areas showing smooth transition of aggregated myocytes orientation across the LV wall in the normal heart and disorganized aggregated myocytes in the HCM heart.

Myocardial disarray is defined as loss of myocyte architectural organization with oblique or perpendicular orientation of myocytes in a disorganized pattern^13^. The orientation of aggregated myocytes has been previously assessed with micro-CT, high-resolution episcopic microscopy (HREM) or Synchrotron imaging using structure tensor analysis (STA)^6,12,21–23^. In our study the voxel resolution was sufficient for determination of the orientation of aggregated myocytes by STA. Areas of disorganized myocytes on micro-CT corresponded to myocardial disarray on histology, and we were able to differentiate HCM from normal hearts based on their myocyte orientation (Fig. 7). Fractional anisotropy was lower in HCM versus normal myocardium, and areas of low FA were matched to areas of myocardial disarray on histology. This was the most consistently different variable between HCM and normal hearts, suggesting that FA quantification may be useful in the postmortem diagnosis of HCM. Recently, FA has been suggested as a useful variable for diagnosis of myocardial disarray in human HCM^24^.

The HA transmural profile was also different between normal and HCM hearts, and abrupt, localized changes in HA and low MDI were associated with myocardial disarray on histology, though the differences between affected and normal hearts were only mild and identified in focal areas of the LV wall.

HCM is a highly heterogeneous disease and to assess the complex pattern of cardiac myocyte orientation we have used different indices that describe different aspects of myocyte orientation. FA showed more marked differences across the LV wall, while HA and MDI showed more localized differences. But these 3 indices should be combined to better assess myocyte disarray and discriminate normal from HCM hearts.

There was no difference in the percentage of circumferential arranged myocytes between normal and HCM hearts. Myocytes have a helical arrangement in the heart with a typical counterclockwise rotation. HA progressively changes from negative angulation in the epicardium to positive angulation in the endocardium. Myocytes in the mid-wall circumferential layer have 0° angulation^21^, and disruption at this site caused by fibrosis and myocardial disarray has been described in HCM hearts^23,24^. This was not observed in our study, but only half of the LV wall in each heart was evaluated.

As with previous pathologic studies in human HCM, there was no correlation between myocardial disarray on micro-CT and aLVM^16^. A recent study showed a weak correlation between LV wall thickness and myocardial disarray in HCM mice using HREM^23^, which could be related to a more detailed assessment of disarray by HREM or differences between HCM in mice versus humans or cats.

We found no association between FA or MDI and myocardial fibrosis, in contrast with a recent DT-MRI study^24^. The differences with the present data may be related to different methods of assessment of fibrosis, or differences between determination of FA with DT-MRI and micro-CT. There are fundamental differences between assessment of myocyte orientation by DT-MRI and micro-CT STA: DT-MRI provides an indirect measurement of myocyte orientation by measuring the direction of water diffusion, which is believed to represent mean myocyte orientation. In STA based methods, the orientation of aggregated myocytes is directly measured, since STA determines their longitudinal direction according to intensity gradients, providing information on the complex 3D arrangement of cardiac myocytes^6,18^. The two methods therefore cannot be directly compared^25^.

In histopathology it is very difficult to completely exclude the presence of myocardial disarray without extensive sampling, and assessment is generally qualitative^15^.

In conventional histopathology it is recommended that the whole circumference of a mid-LV transverse slice is assessed for presence of disarray^13^, but as disarray is regional and not always associated with regions of more severe LVH, it may be missed^15^. Others have suggested that samples are taken at 3 different levels, at the base, mid and apical LV^15,16^, or a minimum of 10 blocks of myocardial tissue^26^, but even then it is challenging and potentially impossible to completely exclude the presence of disarray in the whole LV without very extensive sampling and thorough histologic examination^15^. Thus, routine histopathology is limited in assessing myocardial disarray. Micro-CT might be useful as an objective method for quantification of the extent and distribution of myocardial disarray, or alternatively to guide sampling and histologic examination.

Poor myocardial detail in micro-CT images from some of the hearts precluded more advanced analyses. In 4 hearts the detail was considered inadequate for quantification of fibrosis and in 13 hearts for quantification of the orientation of aggregated myocytes. We have searched extensively for potential causes of decreased myocardial detail, including variations in the scanning protocol or poor postmortem tissue preservation. We rescanned these hearts and changed our scanning settings/protocol by extending the scanning times, increasing scan power and iodination periods to try to improve detail, but to no avail. The cause for reduced image quality in these hearts remains unclear.

### Limitations

This study had several limitations. Contrast agents are required for micro-CT scanning of soft tissues, although iodine does not interfere with histologic analysis^5,8^. A more objective quantification of the histologic lesions would have been preferable, such as unbiased stereological analysis. This could have provided histologic estimations of global LV fibrosis volume, which would have been more accurate for direct comparison to micro-CT determined fibrosis volumes than simple 2D histologic assessment. However, in routine histopathology myocardial disarray and fibrosis are assessed qualitatively or semi-quantitatively, and this study was conducted in clinical cases undergoing routine postmortem exams. Future studies comparing micro-CT with objective quantitative determination of myocardial fibrosis and disarray are necessary to validate our findings.

Micro-CT can quantify cardiac histopathologic lesions but cannot determine diastolic or systolic LV function, so we were unable to assess the degree of LV dysfunction based on the structural changes detected on micro-CT.

Poor myocardial detail in micro-CT images from some of the hearts precluded more advanced analyses. The cause for reduced image quality in these hearts remains unclear.

The micro-CT analyses and pathological examinations were performed by single observers (JNM, PGC and MD), but intra-observer variability was not assessed.

## Conclusions

Our study showed that postmortem micro-CT imaging can detect and quantify the cardinal pathologic features of HCM, including myocardial disarray, which is difficult to quantify with conventional histopathology. Micro-CT therefore has potential value in the postmortem diagnosis of HCM, either as a stand-alone diagnostic modality or as a method of identifying focal lesions to allow targeted sampling for conventional histopathology.

## Methods

This study was approved by the institutional ethical review board of the Royal Veterinary College (RVC, URN 2016-1638-3). The clinical procedures of euthanasia and postmortem examination were done under the Veterinary Surgeons Act as part of recognised veterinary practice. The owners provided informed consent to use the heart tissue obtained for research purposes. The College’s Clinical Research Ethical Review Board scrutinised the consent form and information given to the owner about the project, ensuring they were fully informed of the research to be conducted on tissues obtained at postmortem. This was a prospective observational postmortem study. Ex vivo isolated feline heart specimens were evaluated by micro-CT at Great Ormond Street Hospital (GOSH), and gross and histopathology examinations were performed by a board-certified veterinary pathologist as the gold standard.

### Sample description

All samples were from pet cats that had died or been euthanized for medical reasons with owner-informed consent. The control group consisted of normal hearts from cats undergoing routine necropsy by the RVC Pathology Service. The HCM group consisted of hearts from clinical cases of HCM diagnosed by veterinary cardiologists.

### Heart preparation

The hearts were promptly excised after natural death or euthanasia with IV pentobarbital sodium, and gently flushed with water to remove clots. Hearts were fixed in 10% formaldehyde solution at a tissue:volume ratio of 1:20 for > 48 h, then weighed before immersion in 2.5% potassium tri-iodide (I_2_KI) with a tissue:volume ratio of 1:30 at room temperature for > 48 h^11^. After iodination, the hearts were washed in water to remove surface contrast medium and towel-dried.

### Micro-CT scanning

Micro-CT scans were performed using a Nikon XTH225 ST or a Med-X microfocus-CT scanner (Nikon Metrology, Tring, UK). The hearts were wrapped with parafilm M (Bemis, Oshkosh, USA) and mounted on a low-attenuation carbon fiber rod to minimize artifacts caused by movement and dehydration during the scan.

One micro-CT scan of the whole heart was obtained followed by scans of 3 transverse sections of the LV. After the initial whole heart scan, the hearts were sectioned longitudinally to correspond with a long-axis 5-chamber echocardiographic view, and 4 transverse ventricular sections were made at 5 mm intervals from the level of the atrioventricular groove to the apex in one half of the heart (half-LV transverse sections) (Supplementary Fig. SI). The most apical ventricular transverse section was discarded, as this was very small in some hearts hindering proper histologic assessment. The other 3 half-transverse sections were individually re-scanned (1 basilar and 2 mid-ventricular sections, coded as T1, T2 and T3 from most basilar to apical, respectively).

Whole heart scans were acquired using individually optimized factors, with an X-ray energy of 80–110 kV and current of 112–178 µA. Exposure time was 708 ms, with an optimized number of projections for each examination and 2 frames per projection, and a detector gain of 24 dB. A tungsten target was used. Scans of the individual LV sections were acquired at 80–90 kV and 111–150 µA. Exposure time was 708 ms, with an optimized number of projections for each examination and 4 frames per projection, and a detector gain of 24 dB. A Molybdenum target was used.

Reconstructions were carried out using modified Feldkamp filtered back projection algorithms with proprietary software (CTPro3D; Nikon Metrology) and post-processed using VGSTUDIO MAX (Volume Graphics GmbH, Germany). Whole heart and LV sections micro-CT scans were reconstructed isotropically. Transverse and longitudinal ‘virtual’ dissections were created from the whole heart scans, corresponding with short-axis and long-axis echocardiographic planes, respectively. This resulted in transverse and longitudinal image stacks of an average of 1485 images per stack for the whole heart scans, and 565 images for each LV section stack. Isotropic voxel sizes ranged between 19 and 24 µm for the whole heart scans and 13–21 µm for the LV sections. All micro-CT data sets were initially viewed using VGSTUDIO MAX and Fiji (2.0.0-rc-67/1.52c) for assessment of image quality and evaluation of cardiac morphology.

### Image analyses

The 3 main pathologic characteristics for HCM diagnosis (increased LVM, presence of fibrosis, and myocardial disarray) were evaluated using the micro-CT data and compared between normal and HCM hearts. The results were compared with gross pathology and histopathology.

### Determination of LVM

Whole heart scan datasets were imported into Fiji as a data sequence and viewed as an image sequence and in 3D. The LV region of interest (ROI) was segmented using short-axis reconstructions of the whole heart scans by manually tracing the endocardial and epicardial borders of the LV myocardium in 1 slice every 5–10 slices using the ’brush’ tool. The most basal slice (defined as the first basilar slice where LV myocardium was present) and the most apical slices (where no intracavitary blood was present) were also included in the mass calculation by tracing only the epicardial border. Papillary muscles and LV trabeculations were included in the ROI and therefore in the LVM calculation. The slices between the manually traced segmentations were automatically traced by applying the interpolation function. In each case, 3D reconstructions of the ROIs (LV segmentation) were created to assess whether segmentation was adequate. LV segmentation for determination of LVM by micro-CT was performed by a single observer (JNM).

### Validation of LVM determination by micro-CT

LVM was initially calculated in 13 representative hearts by micro-CT using 2 different methods and each compared with the actual LVM (aLVM) obtained by weighing the hearts. The method with better agreement with aLVM was then used for subsequent LVM determination. Method 1 (M1-Fiji) used the open source image processing software, Fiji, to calculate the LV volume by multiplying the area of each LV slice by voxel size and sum the volumes of all slices. Method 2 (M2-Imaris) used the software package Imaris (Bitplane AG), to calculate surface area and volume from 3D reconstructions of the LV (automated software). LVM was obtained in both methods by multiplying total LV volume by myocardial density (1.05 g/ml)^27^.

Myocardial density is typically assumed to be 1.05 g/ml^28^, but as the studied hearts were stained with iodine, myocardial density was assessed to determine whether iodine had changed the tissue density. Twelve cubes of LV myocardial tissue (≈ 4 mm) without endocardial and epicardial surfaces, as described^28^, were cut from 2 different hearts. The volume of each myocardial section was calculated by water displacement method using a graduated cylinder and its mass measured in a digital balance. Myocardial density was calculated as mass/volume. The LV myocardial density in 12 sections of iodinated hearts was 1.05 (SD 0.15) g/ml.

Actual LVM (aLVM) was determined after the initial micro-CT scan by weighing the LV after dissecting the great vessels, atria and right ventricular free wall. The LV was then weighed 5 times on a digital balance and the mean defined as aLVM.

### Quantification of myocardial fibrosis in HCM hearts

LV myocardial fibrosis was quantified in the HCM whole heart and LV section micro-CT datasets. In the LV sections, the micro-CT results were compared with the histologic assessment.

The datasets were viewed and cropped in Fiji and then imported to Imaris for segmentation and quantification of LV fibrosis. Fibrosis was differentiated from normal tissue using semi-automated segmentation^5^ based on variation in gray-scale values between normal and fibrotic tissue. A preliminary visual inspection of micro-CT images and their equivalent histologic LV transverse sections showed that hypodense areas in micro-CT appeared to correspond with fibrosis on histology, reflecting different X-ray attenuation between normal myocardium and fibrosis. Hypodense areas of similar gray-scale values across the entire specimen were considered to represent fibrosis and were segmented and quantified by a semi-automated region growing method by image processing software (Imaris)^5^. The ‘magic wand’ tool was used to manually segment the ROI (hypodense/fibrotic areas) in each 10th slice from LV base to apex in the whole heart scans and from the first to last slice in the LV section images. Segmentation tools combined the manually segmented areas by intensity of pixels (voxels) creating a 3D surface, and the volume of the 3D reconstruction representing fibrosis was calculated (Fig. 1A). LV segmentation for determination of myocardial fibrosis by micro-CT was performed by a single observer (JNM).

### Quantification of the orientation of aggregated myocytes

The orientation of aggregated myocytes was automatically quantified in T2 sections by a single observer (PGC). The datasets were viewed and cropped in Fiji, then imported to Seg3D (v2.4.0 RC 3) for semi-automated LV segmentation, excluding the right ventricle and papillary muscles. Only the LV walls were included in the orientation calculations.

A gradient structure tensor method implemented in Matlab (MathWorks Inc., Natick, USA, R2018a) was used to assess the orientation of aggregated myocytes in a mid-LV slice (section T2) as previously described^21–23^. Briefly, image intensity gradient in x, y and z directions was calculated for each image voxel using a central difference algorithm. The structure tensor was obtained as the cross product of gradient vectors. Eigen-decomposition was then applied to the structure tensor to obtain the three eigenvalues (λ_1,_ λ_2,_ λ_3_) and eigenvectors (v_1,_ v_2_, v_3_). The vector aligned with the long axis of myocytes was that with the smallest eigenvalue (tertiary eigenvector, v_3_), representing the direction with least intensity variation. The helical (or inclination) angle (HA) of the aggregated myocytes was calculated as the angle between the tertiary eigenvector (v_3_) and the local circumferential plane defined by the local transmural and circumferential directions (Supplementary Fig. SII)^23^. Besides HA, other parameters were calculated to assess orientation more broadly: fractional anisotropy (FA), myocardial disarray index (MDI) and percentage of circumferential arranged myocytes^23^.

FA was calculated as:

$$FA = \sqrt {\frac{3}{2}} \frac{{\sqrt {\left( {\lambda _{1} - \widehat{\lambda }} \right)^{2} + \left( {\lambda _{2} - \widehat{\lambda }} \right)^{2} + \left( {\lambda _{3} - \widehat{\lambda }} \right)^{2} } }}{{\sqrt {\lambda _{1}^{2} + \lambda _{2}^{2} + \lambda _{3}^{2} } }},$$

where $$\widehat{\lambda } = \frac{{\lambda_{1} + \lambda_{2} + \lambda_{3} }}{3}.$$

In diffusion tensor MRI (DT-MRI), FA quantifies the degree of water diffusion anisotropy. FA values range between 0 and 1, where values close to 1 represent ‘restricted water diffusion’ and values near 0 represent ‘unrestricted diffusion’^24^. Similarly to DT-MRI, FA calculated from the structure tensor quantifies the degree of anisotropy in the direction of aggregated myocytes. FA values close to 1 reflect myocytes aligned in the same direction, while values near 0 represent myocytes arranged in random directions.

The MDI quantifies for each image voxel the angular uniformity (or collinearity) of the longitudinal direction of aggregated myocytes, represented by the tertiary eigenvector (v_3_) within a neighborhood that consists of the voxel itself and its nearest neighbors^23^. The computation of MDI was performed using a ROI of 15 × 15 × 15 voxels with values ranging from 0 to 1. A MDI value close to 1 at a given voxel indicates that the myocardial disarray is negligible in the neighborhood of this voxel, whereas values of MDI close to 0 denote a high degree of myocardial disarray (Supplementary Fig. SII)^23^.

Each orientation parameter (HA, FA and MDI) was assessed in the whole T2 LV section and in 5 different LV segments (ROIs): septal, posterior-septal, posterior, posterior-lateral and lateral (Supplementary Fig. SIII). Histograms of HA, MDI and FA were computed for the whole T2 LV section and each of the 5 LV segments. Additionally, the HA transmural profile (from endo- to epicardium or right-side endocardium) was computed for each of the 5 LV segments.

### Pathologic examination

After scanning, hearts were immersed in 4% sodium thiosulphate for 48 h to remove the iodine and then returned to 10% formaldehyde solution before sectioning. The longitudinal LV section and the 3 LV transverse sections were paraffin-embedded, sectioned at a thickness of 4 µm and stained with hematoxylin and eosin, and Masson’s trichrome.

**Figure 8:**
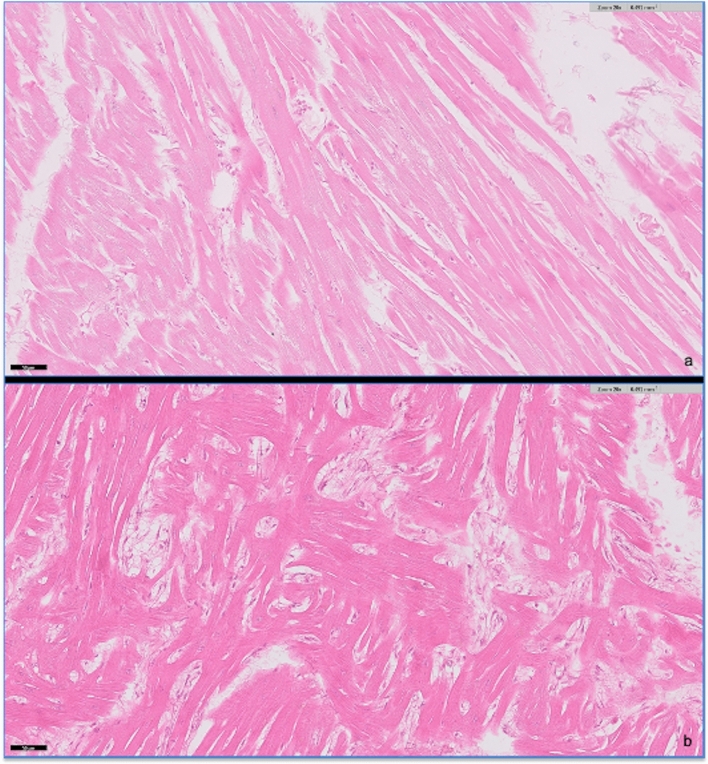
Myocardial disarray was defined on histology as absent (**a**) or present (**b**). Myocardial disarray is defined as loss of myocyte architectural organization characterized by myocytes being obliquely or perpendicular oriented to each other in a bizarre and disorganized pattern.

Gross and histopathology examinations were performed by a single pathologist. The final cardiac postmortem diagnosis was made while blinded to the micro-CT findings. HCM was defined as increased heart weight (> 20 g) associated with LVH and myocyte hypertrophy, myocardial disarray and interstitial/replacement fibrosis^14^. Interstitial fibrosis was defined as an increase in collagen located between myofibers, which does not replace or entrap myofibers, and presents as individual fine stands of collagen with a regular pattern of distribution, arranged in parallel to the myofibers and not generally interconnecting. Replacement fibrosis was defined as an increase in collagen which replaces lost (necrotic) myofibers. It may run in multiple directions, with a haphazard arrangement, may isolate and entrap individual myofibers if more severe, or be present as uninterrupted fibrosis. It tends to be more focal/multifocal. Histopathologic lesions of replacement fibrosis were scored as mild, moderate or severe using a semi-quantitative scoring system (Table 1). Myocardial disarray was scored as present or absent (Fig. 8). Areas of disarray at the junction of the right ventricle with the septum, adjacent to blood vessels or at areas of extensive replacement fibrosis were not considered true disarray^13,15,29^, and were not quantified. Unblinding occurred after the initial micro-CT imaging analyses in order to perform histologic comparison with the micro-CT findings.

**Table 1:** Histopathologic lesions of replacement fibrosis were scored using a semi-quantitative scoring system (histology images stained with Masson’s trichrome, × 10 magnification).

Semi-quantitative scoring system of replacement fibrosis		
Mild	Fibrosis incorporates small areas with apparent individual/small numbers of myocytes lost; black arrows indicate replacement fibrosis and green arrows demonstrate interstitial-type fibrosis	
Moderate	Fibrosis incorporates larger areas with apparent loss of multiple myofiber in adjacent areas, and with encircling and entrapment of surrounding myofibers by bands of fibrous tissues; myofibers may be individualized by the fibrous tissues	
Severe	Fibrosis occupies larger areas with loss of larger numbers of often adjacent myofiber, with complete replacement of myofiber by contiguous areas of fibrous tissues, forming scars

### Histologic comparisons

Histologic comparisons with micro-CT findings were performed by comparing micro-CT images of each LV section with the corresponding histology section. LV section scan datasets (T1–T3 of each heart) were imported into Fiji and viewed as a sequence of images to match the histology section as closely as possible (Figs. 1, 3). Histologic comparisons with micro-CT were focused on identifying and documenting segments of myocardial fibrosis and disarray in stained sections with Masson’s trichrome and H&E, respectively.

### Statistical analysis

Data were tested for normality graphically and by Shapiro–Wilk test. Normally distributed data are reported as mean (± standard deviation) and non-normally distributed data as median [range]. Comparisons between aLVM, M1 and M2 were made by repeated-measures ANOVA with Tukey’s post-hoc test. Bland–Altman analysis was used to assess agreement between LVM determined by micro-CT and aLVM.

The association between volume of fibrosis determined by micro-CT and the semi-quantitative histologic assessment of fibrosis was done by comparing micro-CT fibrosis values between histology groups (absent/mild vs moderate or absent/mild vs moderate/severe) in each LV section (T1, T2 and T3) by Mann–Whitney U-test. Median fibrosis volume between the 3 LV sections was compared by Friedman test with Dunn’s post-hoc test. Pearson’s correlation coefficient was performed to assess the association of FA and MDI with heart weight, aLVM and fibrosis. HA, FA and MDI were compared between normal and HCM hearts as the mean in each LV segment and whole LV T2 section. The distribution of values of HA, FA and MDI at each LV segment was also assessed, with differences across segments (septal, posterior-septal, posterior, posterior-lateral and lateral) between normal and HCM groups tested using a two-sample Kolmogorov–Smirnov test. The FA and MDI values were compared between groups for the whole LV T2 section by Students’ independent t-test, and between each of the 5 LV segments by repeated measures ANOVA. The latter was run for each variable (FA and MDI) including group, segment and their interaction in the model with LSD post-hoc test. The mean HA transmural profiles for normal and HCM groups were compared at each relative position across the LV wall for each LV segment by Mann–Whitney U-test. A linear regression fitting of the HA transmural profiles at each LV segment was also performed and the slope (B1) and linearity (R^2^) compared between normal and HCM groups.

P values < 0.05 were considered statistically significant. Statistical analysis was performed using SPSS (v25-26) and GraphPad Prism 7.

## Supplementary information

Supplementary Information.

## Data Availability

The datasets generated during and/or analyzed during the current study are available from the corresponding author on reasonable request.
