# Supplementary material for: Micro-computed tomography (micro-CT) for the assessment of myocardial disarray, fibrosis and ventricular mass in a feline model of hypertrophic cardiomyopathy
Source: Sci Rep. 2020 Nov 19;10:20169. doi: 10.1038/s41598-020-76809-5 (PMC7678873; doi:10.1038/s41598-020-76809-5)

**SUPPLEMENTAL INFORMATION**

**Micro-computed tomography (micro-CT) for the assessment of myocardial disarray, fibrosis and ventricular mass in a feline model of hypertrophic cardiomyopathy**

Jose Novo Matos, PhD; Patricia Garcia-Canadilla, PhD; Ian C. Simcock, MSc; J. Ciaran Hutchinson, PhD; Melanie Dobromylskyj, PhD; Anna Guy, MRes; Owen J. Arthurs, PhD; Andrew C. Cook, PhD; Virginia Luis Fuentes, PhD

**Supplementary Figure I** Micro-CT scanning and pathology protocol. Initially a whole heart micro-CT scan was performed followed by scans of 3 LV sections. The heart specimens were scanned on micro-CT twice, first as a whole heart scan and secondly transverse ventricular sections were scanned individually. After micro-CT scans longitudinal and transverse LV sections were submitted for histology.

**
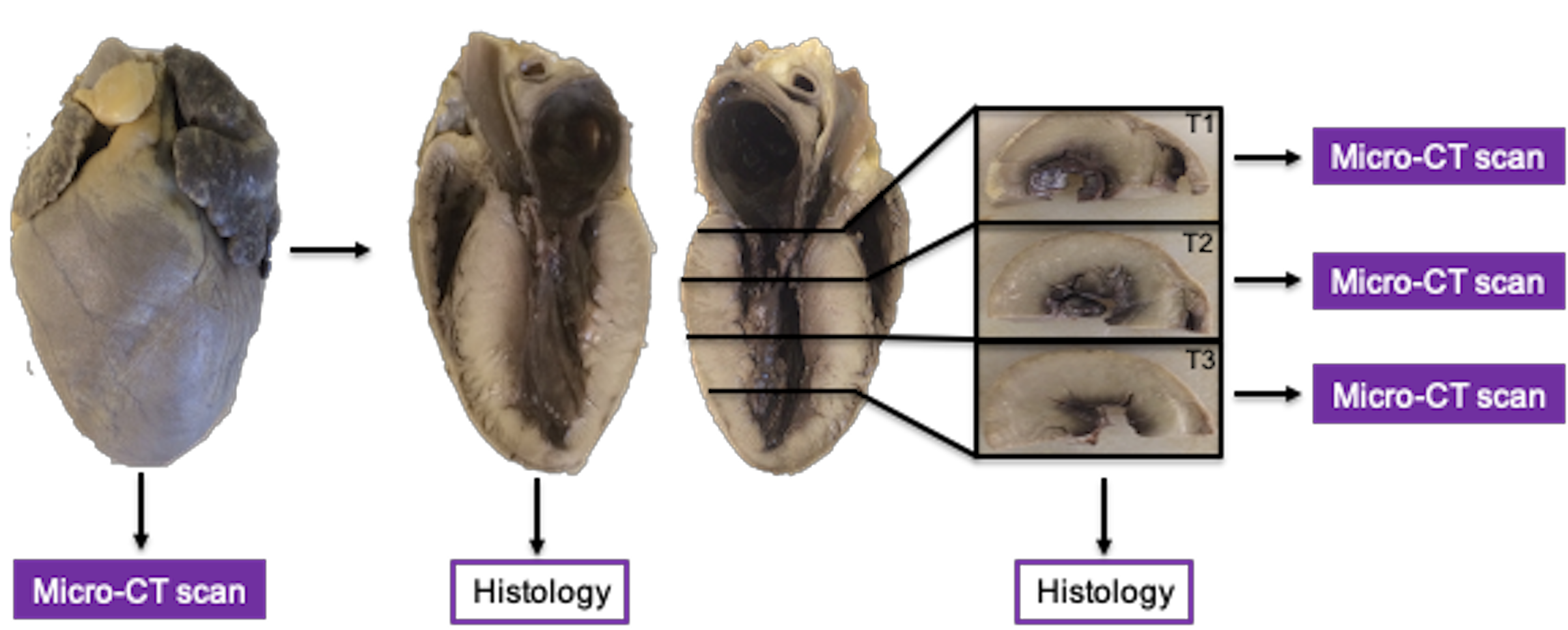
**

**Supplementary Figure II** a. Local coordinate system and definition of helical angle (⍺_H_), calculated as the angle between the tertiary eigenvector (v_3_) and the local and the local circumferential plane defined by the local radial ($\vec{r}$) and circumferential ($\vec{c}$) directions. b. Schematic representation of the region of interest (ROI) defined to calculate the structure tensor in a given voxel, formed by the voxel itself and their 8 nearest voxels. In this drawing, each cylinder represents a single cardiomyocyte. The mid panel illustrates the eigenvector system ($\vec{v}_{i}$, i=1…3) and corresponding ellipsoids obtained with structure tensor analysis in healthy (normal) vs. hypertrophic cardiomyopathy (disarray) showing high anisotropy represented by a flat ellipsoid (organised myocardium) vs. low anisotropy represented by a more spherical ellipsoid (disorganised myocardium). The right panel illustrates the calculation of the myoarchitectural disarray index (MDI) in the same ROI used to calculate the structure tensor. In this drawing we have plot the tertiary eigenvectors in the 2D plane indicated in grey in the right panel. MDI in a given voxel quantifies the angular uniformity between the tertiary eigenvectors of the voxel itself (red square) and the tertiary eigenvectors of their nearest neighbours. In heathy cardiac tissue, all the eigenvectors have similar orientations, while when disarray is present, all the eigenvectors have very different orientations and therefore MDI is close to 0.^23^

**
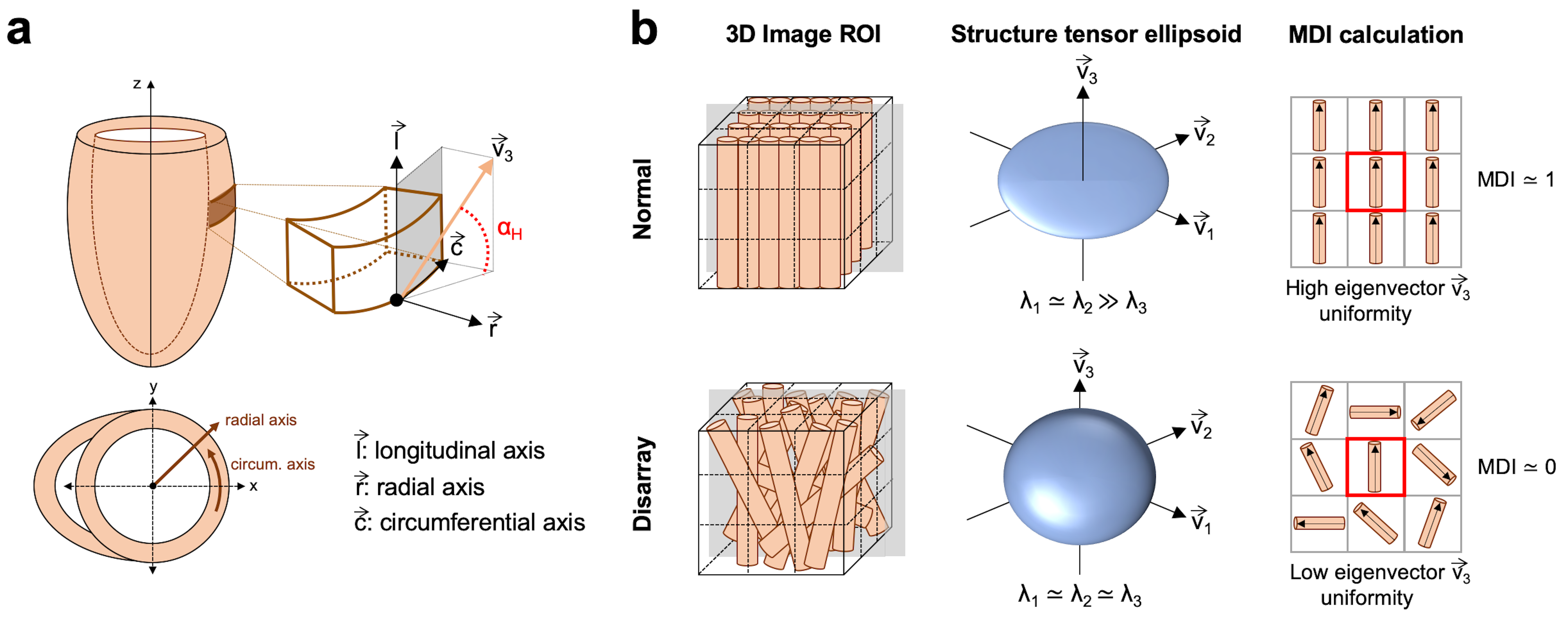
**

**Supplementary Figure III** Myocyte aggregates orientation was assessed in a mid-LV section (T2) in 5 different segments: septum, posterior-septal, posterior, posterior-lateral and lateral


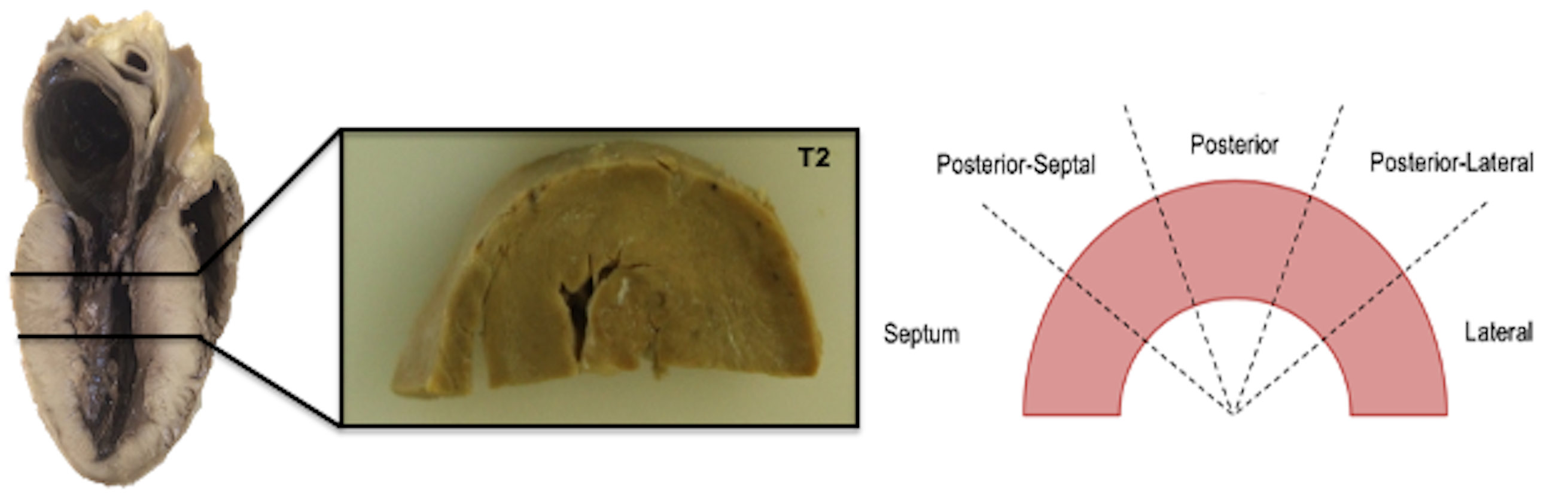

Supplement: Supplementary file 1 — Supplementary Information. [file 41598_2020_76809_MOESM1_ESM.docx]
